# Microalgae Cultivation on Anaerobic Digestate of Municipal Wastewater, Sewage Sludge and Agro-Waste

**DOI:** 10.3390/ijms17101692

**Published:** 2016-10-10

**Authors:** Luca Zuliani, Nicola Frison, Aleksandra Jelic, Francesco Fatone, David Bolzonella, Matteo Ballottari

**Affiliations:** Dipartimento di Biotecnologie, Università di Verona, Strada le Grazie 15, 37134 Verona, Italy; zuliani.luca@outlook.com (L.Z.); nicola.frison@univr.it (N.F.); alexsandra.jelic@gmail.com (A.J.); francesco.fatone@univr.it (F.F.); david.bolzonella@univr.it (D.B.)

**Keywords:** microalgae, anaerobic digestate, biofuels, photosynthesis

## Abstract

Microalgae are fast-growing photosynthetic organisms which have the potential to be exploited as an alternative source of liquid fuels to meet growing global energy demand. The cultivation of microalgae, however, still needs to be improved in order to reduce the cost of the biomass produced. Among the major costs encountered for algal cultivation are the costs for nutrients such as CO_2_, nitrogen and phosphorous. In this work, therefore, different microalgal strains were cultivated using as nutrient sources three different anaerobic digestates deriving from municipal wastewater, sewage sludge or agro-waste treatment plants. In particular, anaerobic digestates deriving from agro-waste or sewage sludge treatment induced a more than 300% increase in lipid production per volume in *Chlorella vulgaris* cultures grown in a closed photobioreactor, and a strong increase in carotenoid accumulation in different microalgae species. Conversely, a digestate originating from a pilot scale anaerobic upflow sludge blanket (UASB) was used to increase biomass production when added to an artificial nutrient-supplemented medium. The results herein demonstrate the possibility of improving biomass accumulation or lipid production using different anaerobic digestates.

## 1. Introduction

Microalgae are unicellular photosynthetic organisms; from an industrial and economic perspective, their cultivation has a great deal of potential [1,2]. Indeed, several valuable products may be obtained from their biomass, ranging from animal and human nutrition, cosmetics and pharmaceuticals, to biofuels such as biodiesel, biogas and hydrogen [3,4,5,6,7,8,9,10]. Liquid biofuels are currently produced using oil crops, including corn and soy, or from sugar cane; however, their adoption on a global scale seems to be unsustainable in terms of required cultivated area and transformation yield [7,11,12]. The large amount of storable lipids and CO_2_ absorption, in addition to the high growth rate without creating competition for arable land, make microalgae the best candidates for biofuel production [7,13,14] or at least economically interesting for compounds such as astaxanthin, vitamins, polyunsaturated fatty acids and pharmaceuticals [15]. The model organism for green algae is *Chlamydomonas reinhardtii* as its genome has already been sequenced and characterized. *C. reinhardtii* is characterized by a ~10 µm cell, two flagella and a large chloroplast. *C. reinhardtii*, however, is rarely used at the industrial level, where more robust and fast-growing species are preferred, such as those belonging to the *Chlorella*, *Scenedesmus* or *Nannochloropsis* genera. The *Scenedesmus* genus counts 74 algal species, typically living in freshwaters as non-motile colonies. Cell morphology varies on a per species basis. For example, *Scenedesmus obliquus* accumulates a high level of lipids in nitrogen deficiency [16] and has been commonly proposed as a candidate strain to treat wastewaters [17,18] and to produce biodiesel [19]. *Chlorella vulgaris* and *Chlorella sorokiniana* are widely cultured to produce food and biofuels as well [20,21]. Their cells are spherical, ranging from 2 to 5 µm, with a thin cell wall and a single chloroplast. They are capable of both autotrophic and heterotrophic growth whenever a proper carbon source is supplied [22]. *Nannochloropsis* species are marine microalgae with high lipid productivity; indeed, *Nannochloropsis gaditana* can store up to 70% of its biomass in oleaginous form [12,23,24]. *Nannochloropsis* cells are non-motile and have a diameter varying from 2 to 8 µm. Microalgal cultivation requires light, CO_2_, and nutrients, such as nitrogen and phosphorus sources, together with different microelements [25,26]. The price of nutrients for cultivation of microalgae is one factor behind the high cost of algae-derived biomass, thereby limiting industrial cultivation of these organisms. Wastewaters and their high nutrient content appear to be a possible solution to obtain nutrients at a low cost, suggesting the possibility of coupling biofuel production with wastewater treatment [2,27,28]. *S. obliquus* was reported in the literature to reach a record of 98% of phosphorus and 100% of nitrogenous component utilization [29]. Biological treatment of wastewaters, agro-waste and sludge, operated at the industrial level, is based on the same capability of self-depuration of a natural water body and can be conducted in aerobic or anaerobic conditions. Anaerobic digestion of wastewater, sludge and agro-waste is commonly used for organic matter stabilization and biogas production [30,31,32], leaving a residual digestate that can be used for fertilizing. Many microalgal species can efficiently grow in these media, stabilizing them without determining waste or by-product production [28].

The aim of this project was to evaluate the capability of different algal strains to exploit waste products (nutrients) resulting from anaerobic digestion of municipal wastewater, sludge and agro-waste from three different treatment plants. Subsequently, possible solutions to reduce costs of microalgal cultivation by exploiting waste-derived substrates can be identified. The tested algal strains include *C. reinhardtii*, *C. vulgaris*, *C. sorokiniana*, *N. gaditana* and two locally isolated *Scenedesmus* strains, referred to as *Scenedesmus* *I* and *II*.

## 2. Results

### 2.1. Production of Anaerobic Digestates

Three different anaerobic digestates were chosen to be tested for microalgal cultivation with different origins and compositions, here referred to as dA, dB and dC. dA originated from a full-scale farm plant where manure of bulls and cows was co-treated together with energy crops (maize and triticale silage). In contrast, dB was produced from the anaerobic digestion of 400–450 m^3^/day of primary and waste activated sludge at mesophilic conditions (37 °C) in the municipal wastewater treatment plant of Verona (Italy). Finally, dC originated from the anaerobic treatment of the municipal wastewater in a pilot-scale anaerobic upflow sludge blanket (UASB) with 16 liters of working volume. Every day, the UASB treated up to 50 liters of municipal wastewater from Verona WWTP at 20−25 °C for the removal of the produced organic matter and biogas production [33].

#### Nutrient Composition of Digestates

Table 1 describes nitrogen, ammonia and phosphorus content of the three selected digestates, dA, dB and dC, compared to High Salt (HS) medium, an artificial medium usually adopted for microalgal cultivation in the laboratory. Both dA and dB presented a relatively high total solid (TS) content, among which 70% and 53% for dA and dB, respectively, resulted in organic form (detected as total volatile solid, TVS). In the case of dC, the TS content was extremely low—Less than 0.1 g/L—while in the case of HS, a TS content of 2 g/L was detected, as it was related to inorganic substances, since TVS content was below the limit of detection. Considering the chemical oxygen demand (COD), TS and TVS values, it is possible to conclude that by removal of TS by centrifugation and filtration, as described in Section 4, most of the carbon organic compounds were removed. Measurement of volatile fatty acids demonstrated that acetic acid was the only carboxylic acid detected at quite low concentrations in all digestates (<100 mg/L in dA and dB, <20 mg/L in dC). dA was the most nutrient-rich substrate among the three tested, with nitrogen and phosphorus levels having increased, respectively, by 28 and almost four times compared to HS. Despite a high phosphorus content, the phosphate concentration was only 1.7% of the total, indicating that most of the phosphorus was not available for algal cultivation and likely in aggregated form. In the case of nitrogen, the ammonium concentration was 56% of the total nitrogen content, more than 26 times the ammonium concentration available in the HS medium. dB digestate presented a nitrogen content 15 times greater than HS due to its being 52% ammonium. The phosphorus concentration was quite similar in HS and dB, even if the phosphate concentration in the latter was only 6.9% compared to HS. It must should also be noted that the phosphorus in the HS medium is extremely high since the phosphate buffer is used to maintain the pH value at around 7.0: other media commonly used for algal cultivation, such as TAP or BG-11, present a phosphate content reduced to 7.5% and 18.5% compared to HS, which is sufficient to prevent phosphorus starvation in microalgae. dC resulted in a substrate with a very diluted content of nutrients; there was a 79.9% decrease of nitrogen content (as it is almost only present as ammonium) and a 99.5% decrease of phosphorus content (as it is almost only present as orthophosphate). The pH value of the different digestates was measured ranging from 7.6 (dC) to 8.2 (dA). The alkalinity of the digestates was more than ten times higher in dB compared to dC and HS, and even higher in the case of dA. The dA digestate appeared to be very dense and turbid, implying reduced light availability causing light limitation for algal growth. In order to better investigate this point, the optical density (OD) of the different digestates was measured at 440 and 680 nm, the two wavelengths at which chlorophyll binds to photosynthetic complexes and shows their maximum absorption. OD measurement was performed after removal of the solid phase by centrifugation and filtration as described in Section 4: dA was characterized by a high OD at both 440 and 680 nm, while much lower OD was measured for dB (Table 1). dC and HS, on the other hand, had almost no absorption at 440 and 680 nm (OD < 0.01). The OD values obtained indicate that in the case of dA and dB ~100%/~90% and 37%/20% of the light would be filtered in 1 cm at 440/680 nm, respectively, while less than 1.3% will be filtered by dC and less than 0.4% in the case of HS. These results indicate that a dA and likely dB must be diluted in order to be used for algal cultivation.

### 2.2. Growth of Micoralgae in Solid Medium

The tested algal strains include the green algae *C. reinhardtii*, *C. vulgaris* and *C. sorokiniana*; the marine species *N. gaditana*; and two different *Scenedesmus* strains which were isolated in Verona, referred to as *Scenedesmus* *I* and *II*. The identification of isolated strains with *Scenedesmus* species was performed from the morphology of the cells as reported in the Supplementary Materials, Figure S1. *N. gaditana* was found to be the species with the smallest cell, with an average diameter of ~1 µm, while *C. reinhardtii* was found to be the species with the largest cells (~10 µm). *C. vulgaris* and *C. sorokiniana* were characterized by an intermediate cell size (~1.5–2 µm), whereas both *Scenedesmus* strains presented an average cell diameter of ~4 µm.

In order to test the possibility of using the three different anaerobic digestates, dA, dB and dC, for algal cultivation, a first growth experiment was conducted on solid medium upon agar addition. The three digestates were tested in different concentrations using either water or HS medium for dilutions (5, 10 and 30 times). Since substrate dC has a reduced nutrient concentration compared to HS, dA and dB, it was used undiluted. Five microliters of three different cell concentrations (10^6^, 10^5^, 10^4^ cell/mL) were spotted onto the solid media and incubated at 25 °C at 80 µmol·m^−2^·s^−1^. Growth of the microalgae strains in the different conditions are reported in the Supplementary Materials, Figure S2.

*N. gaditana* did not grow on plates with dA or dB, and developed tardily with dC diluted 5 times in HS. These results indicate that the nutrient composition and/or the salinity of tested conditions are not sufficient to sustain *N. gaditana* growth. *C. reinhardtii* showed a reduced growth in every condition in the presence of anaerobic digestates, while *C. vulgaris*, *C.* *sorokiniana*, *Scenedesmus* *I* and *II* developed readily in most of the conditions tested.

It is important to note, however, that *C. vulgaris*, *C.* *sorokiniana*, and *Scenedesmus* cells plated in the presence of substrate dA were characterized by a retarded growth, probably due to its strong color that may reduce light availability (see OD values in Table 1). Indeed, when the substrate dA was diluted 30 times, the growth of *C. vulgaris*, *C. sorokiniana*, and *Scenedesmus* strains was enhanced compared to the growth in HS or compared to the growth in dA diluted five times only. Growth in substrate dB proceeded at a high rate with the exception of *Scenedesmus* colonies when grown in dB diluted 30 times in water, which induced a yellow-orange color of colonies on plates, likely due to nutrient-related stress inducing chlorophyll degradation and carotenoid accumulation. Growth in plates prepared with dC undiluted or diluted with water, showed a significant reduction in algal growth in all cases, with again the yellow-orange coloration in the case of *Scenedesmus* colonies. Since dC digestate is the substrate least enriched in nutrients, this reduced growth and carotenoid accumulation is likely due to a reduction of nutrients to a level that does not sustain algal development. In support of this hypothesis, when dC was diluted with HS, the growth of *Scenedesmus* and *Chlorella* strains was significantly increased. Results obtained in plates demonstrate that *Chlorella* and *Scenedesmus* can be cultivated in the presence of dA or dB even diluted with water, while dC did not sustain algal growth if not added to HS medium, due to nutrient shortage.

### 2.3. Growth Tests in Closed Photobioreactors

**Figure 1:**
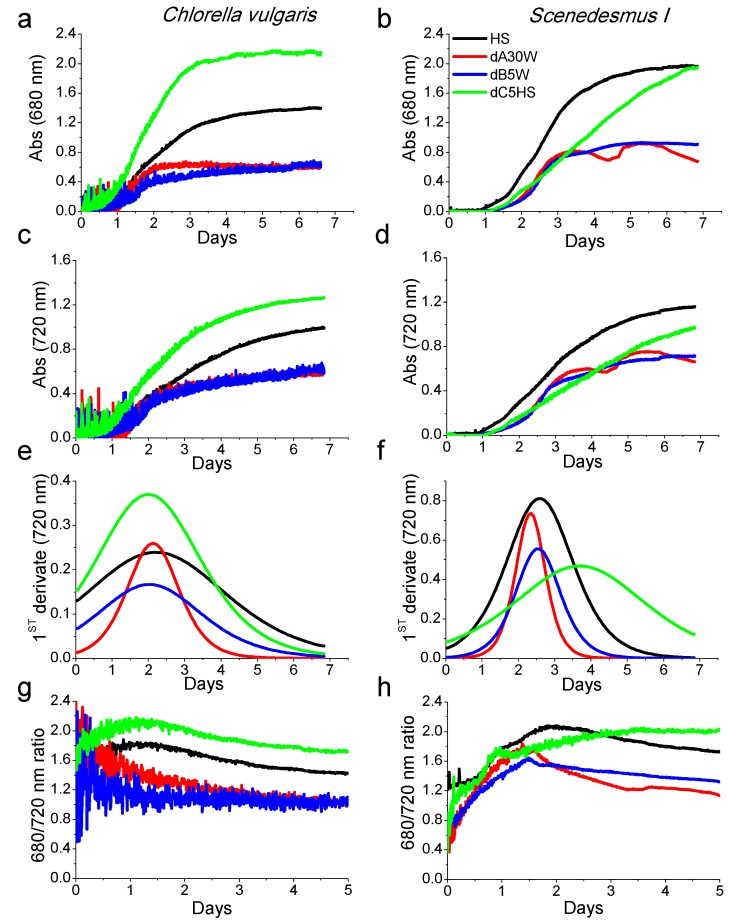
Growth kinetics measured at 680 and 720 nm of *C. vulgaris* and *Scenedesmus I.* (**a**,**c**) growth curves of *C. vulgaris* measured at 680 and 720 nm, respectively; (**b**,**d**) growth curves of *Scenedesmus I* measured at 680 and 730 nm, respectively; (**e**,**f**) first derivative of 720 nm growth kinetics; and (**g**,**h**) ratio of 680 and 720 nm absorption during cultivation.

Selected conditions from the previous experiment were further tested using the multicultivator MC 1000 where eight small photobioreactors with total volume of 85 mL can be run in parallel for algal cultivation. Digestate dA was used at its maximum dilution (30 times in water, hereafter called dA30W), in order to limit the light-filtering effect, while dB and dC were diluted five times in water (dB5W) and in HS (dC5HS), respectively, according to the results obtained in solid medium (Figure S2). HS medium was adopted as a standard medium for microalgal cultivation. Organisms chosen for this test were *C. vulgaris* and *Scenedesmus I* because of their excellent growth rate in the previous experiment. Growth conditions were maintained by the instrument itself at 25 °C and 400 µmol·m^−2^·s^−1^ light intensity for six days. Starting inoculum was 2 ×·10^6^ cell/mL. The growth curves in the different conditions were followed measuring the chlorophyll absorption at 680 nm and the cell scattering at 720 nm (Figure 1). In the case of *C. vulgaris*, the growth curves reported in Figure 1 show a more rapid growth and a higher cell accumulation in the presence of substrate dC5HS, compared to other substrates. Contrarily, the growth curves when cells were grown in dA30W or dB5W showed reduced performances compared to the control condition (HS). In the case of *Scenedesmus* *I*, growth kinetics were more rapid in HS medium compared to dC5HS, whereas in the case of *C. vulgaris*, both 680 and 720 nm traces were reduced in dA30W and dB5W compared to HS medium. The daily maximum productivity of the different cultures was estimated as the maximum of the first derivative of the growth kinetics measured at 720 nm and is reported in Figure 1e,f. In the case of *C. vulgaris*, maximum daily productivity was obtained; in the case of d5CHS, HS was the growth medium yielding the highest daily productivity with *Scenedesmus I*. However, it is interesting to note that the daily maximum productivity of dA30W was for both strains similar to the case of cells grown in HS. Figure 1g,h shows the 680/720 ratio, which can be used to follow the changes in the chlorophyll content per cell, which can be decreased due to stress conditions or cell senescence. In the case of *Chorella vulgaris*, decreasing trends of 680/720 ratio were evident for substrates dA30W and dB5W, indicating a continuous reduction of chlorophyll content per cell. Conversely, in the case of *C. vulgaris* grown in HS or in dC5HS, or *Scenedesmus I* grown in dA30W, dB5W or HS, after an initial increase, a reduction of the 680/730 ratio was evident. The 680/730 ratio in the case of *Scenedesmus I* grown in dC diluted in HS showed a continuous increase to saturation, indicating that growth in optimal conditions retards cell senescence.

**Figure 2:**
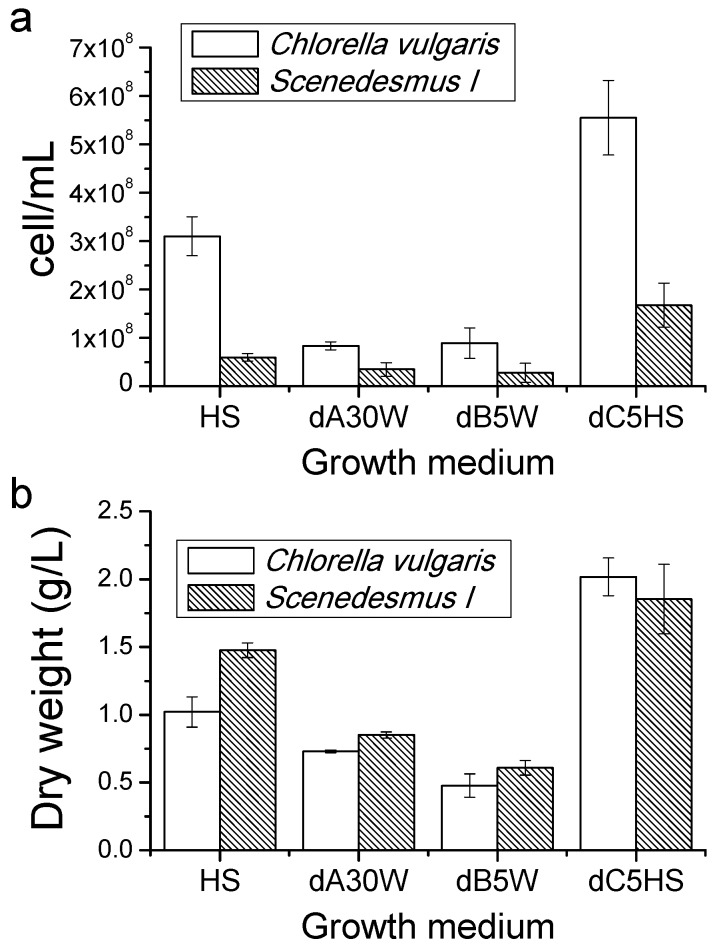
Cell density and dry weight recovered from algae cultures: (**a**) cell density expressed as number of cells per mL; and (**b**) dry weight expressed as g/L.

At the end of the growth curves (Figure 1), cell density and biomass accumulation were measured and the obtained results are reported in Figure 2: Cells grown in substrate dC showed a higher cell density compared to cells grown in HS for both *C. vulgaris* and *Scenedesmus I.* Alternatively, in the case of the substrates dA30W and dB5W, the cell density was reduced.

Dry weight of harvested biomass at the end of growth curves is reported in Figure 2b: increased biomass accumulation was evident for cells grown in substrate dC5HS compared to cells grown in HS only, with a 98% and 25% production increase for *C. vulgaris* and *Scenedesmus I*, respectively. In the case of the growth medium obtained by diluting dA30W, biomass production was reduced by 28% and 43%, respectively, for *C. vulgaris* and *Scenedesmus* *I*, whereas in the case of dB5W, the decline was 53% for *C. vulgaris* and 59% for *Scenedesmus I*. It is important to note that the *Scenedesmus* cells were generally bigger (Supplementary Materials, Figure S1) compared to *C. vulgaris* cells, thereby explaining the similar or even increased dry weight of *Scenedesmus* cultures as compared to *C. vulgaris*, despite the reduced cell concentration.

**Figure 3:**
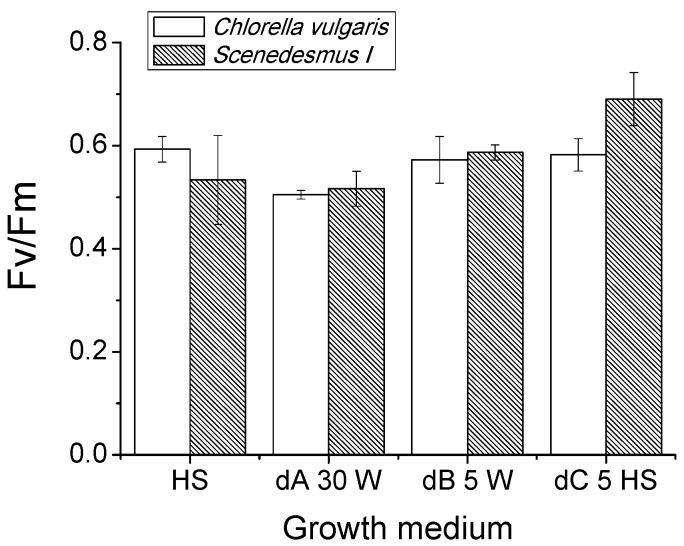
Fv/Fm of algae cultures at the different growth conditions at the end of the exponential phase.

Differences in biomass accumulation in dA30W and dB5W substrates suggest a possible stressing conditions for cells. Quantum efficiency of Photosystem II can be determined by measuring chlorophyll a fluorescence emission through the photosynthetic parameter Fv/Fm. Fv/Fm is a fluorescence parameter commonly used to evaluate the stressing conditions of a photosynthetic organisms, being reduced when photosynthetic apparatus is stressed [34]. In all analyzed conditions, Fv/Fm values ranged between 0.5 and 0.59 (Figure 3), typical values for algae cultures grown in high light conditions [35]. The only exceptions were found in the case of *Scenedesmus I* grew in dC5HS, where Fv/Fm values reached a value of 0.69. This results indicate that growth in the different growth media did not significantly alter the quantum efficiency of PSII, with the exception of substrate dC for *Scenedesmus I* cells which were less stressed by high light compared to cells grown in HS only or in dA30W and dB5W. The reduction of biomass accumulation observed for dA and dB should not be related to a specific destabilization of photosynthetis apparatus, but rather to the availability of the different nutrients.

#### Proteins, Lipids and Pigment Accumulation in Photobioreactor Cultivation

**Figure 4:**
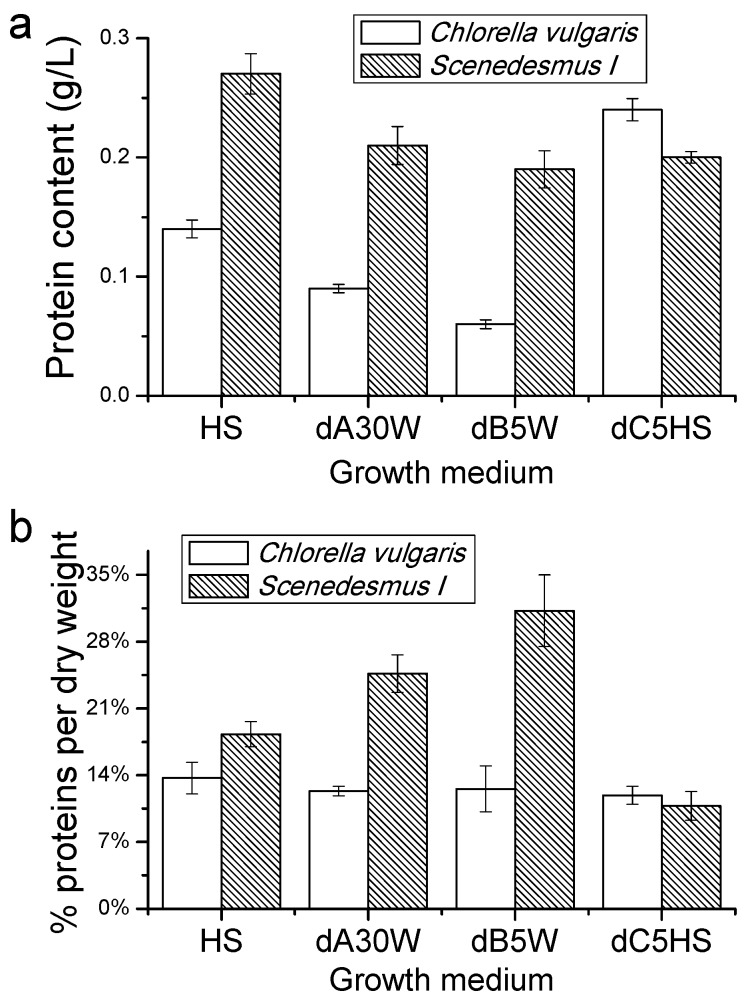
Protein content of algae cultures: (**a**) total protein content (g/L) accumulated in microalgae cultures; and (**b**) percentage of protein content on the overall biomass harvested.

Accumulation of proteins, lipids and pigments by cells of *C. vulgaris* and *Scenedesmus I* grown in photobioreactors were determined at the end of the growth curves. As reported in Figure 4, *C. vulgaris* showed a maximum production of proteins per volume of culture (g/L) when grown in substrate dC5HS, while a strong decrease in total proteins produced by *C. vulgaris* culture grown in dB5W. Differently, no significant change was observed in the protein produced in the *Scenedesmus I* cultures. The percentage of protein content per biomass produced did not significantly changed in *C. vulgaris*, while an increase was evident for *Scenedesmus I* grown in dA30W and dB5W with a maximum level of 31.1% in the latter case. The lowest content of proteins per g of biomass accumulated was instead measured in the case of *Scenedesmus I* grown in dC5HS, with a value of 10.8% of cell biomass being proteins.

**Figure 5:**
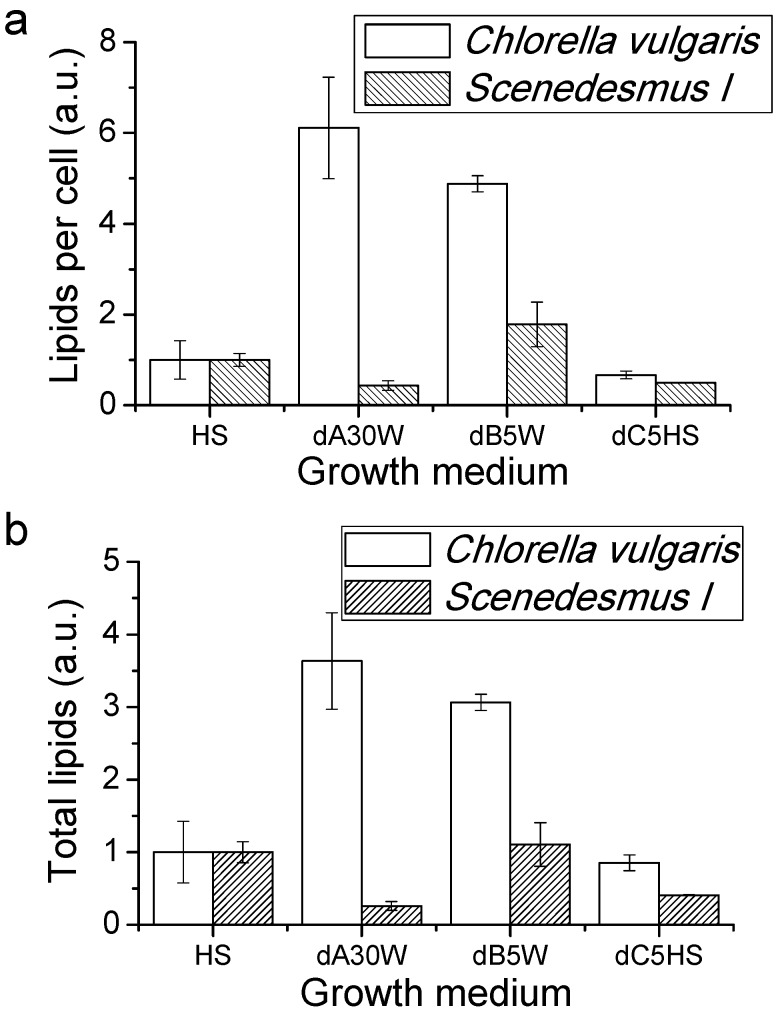
Nile red assay for lipid quantification in microalgae cultures: (**a**) Nile red fluorescence emission normalized per cell content indicating the lipid content per cell; and (**b**) total lipids accumulated on microalgae cultures expressed as Nile red fluorescence emitted by the same volume of culture in the different conditions. The values reported were normalized to the case of cells grown in HS medium and expressed as arbitrary units (a.u.).

Lipid content has been determined by Nile red staining, a probe which fluorescence emission at 575 nm increases in nonpolar environments, a commonly used assay for determination of lipids concentration in microalgae [36]. As shown in Figure 5, *C. vulgaris* grown in substrates dA30W or dB5W, were characterized by a strong increase of lipid content per cell or per total volume of culture compared to cells grown in dC5HS or in HS only. The lipid content increase may be attributable to a nutritional stress condition, resulting in a buildup of triacylglycerides, typically adopted as a reserve molecule to survive in adverse environmental conditions [37]. In contrast, only a 45% increase in lipid content per cell was recorded for *Scenedesmus I* grown in dB diluted in water compared to cells grown in HS, resulting into a similar lipid productivity per volume of culture. *Scenedesmus I* grown in dA30W or dC5HS instead showed a decrease of lipid content per cell and lipid accumulation per volume of culture compared to cells grown in HS.

Pigments extracted by the different cultures were analyzed by HPLC [38]. Samples grown in the presence of substrate dA30W and dB5W showed a noticeable decrease of chlorophyll/carotenoid ratio compared to other growth conditions, indicating a preferential accumulation of carotenoids rather than chlorophylls (Table 2). Biosynthetic pathways of carotenoids and lipids share few metabolic steps in the early stages of their pathways. Considering the results obtained by the Nile red assay, it is possible to state that cells growing is dA30W and dB5W were stressed, thus boosting both lipid and carotenoid accumulation. Interestingly, in the case of dB5W, a strong reduction in the Chl a/b ratio was evident, suggesting in this condition a specific degradation of core complexes (containing only chlorophyll a)—A typical phenomenon observed in developmentally unfavorable conditions.

## 3. Discussion

In this work, different anaerobic digestates were tested as a source of nutrients for microalgal cultivation. As reported in Figure S2, digestates dA, dB and dC could be successfully used for microalgal cultivation in plates, yielding the best results in the cases of the *Chorella* and *Scenedesmsus* species. This result is in agreement with a previous report claiming strains related to these two species were the most efficient in growing in wastewater-derived substrates [39]. Only in the case of *N. gaditana* did it appear that any digestates could sustain algal growth, while previous works reported the possibility of using anaerobically digested municipal wastewater for *N. gaditana* [40] or *Nannochloropsis salina* [41,42] cultivation. The low salinity of the different digestates, especially upon dilution, is likely the main reason for the absence of growth of *N. gaditana*, since in a previous report it was found that the anaerobic digestates investigated were diluted with artificial salt water. We thus cannot exclude that, upon salt addition, *N. gaditana* could be efficiently grown and capable of exploiting the different digestates investigated in this study. On the basis of the results obtained in the plates, *C. vulgaris* and *Scenedesmus I* strains were cultivated in a closed photobioreactor using as growth media diluted digestates and compared to growth in the HS medium. Results show that digestate dC from the UASB reactor cannot sustain algal growth due to the low content of nutrients (Table 1). dC is characterized by a nitrogen content three times lower than artificial minimum medium used commonly in the laboratory for microalgal cultivation (HS), while phosphorous concentration is reduced by 98% compared to HS [43,44], or 85% compared to other artificial media such as TAP or BG-11 [45,46]. However, dC can be used to effectively enrich nutrient content of other growing media; the addition of dC to HS medium in a 1:4 ratio indeed resulted in a 98% and 25% increase in biomass accumulation in the case of *C. vulgaris* and *Scenedesmus I*, respectively. dC can thus be adopted as a nitrogen additive for microalgal cultivation to be added into other media. The presence of an inorganic source for carbon as carbonate in dC can also be considered one of the reasons for the increased biomass accumulation observed compared to the HS medium, where carbonate was not present. Anaerobic digestates herein investigated, dA, dB and dC, were characterized by the presence of an organic carbon source as acetic acid which potentially could be exploited by algae to increase biomass accumulation in a mixotrophic metabolism [47,48,49,50]. *Chlorella*, *Scenedesmus* and *Chlamydomonas* strains indeed were reported to switch to a mixotrophic metabolism in presence of acetate increasing biomass accumulation compared to autotrophic growth [50,51,52]. The presence of carboxylic acids and in particular acetic acid on different anaerobic digestates has been previously reported and correlated with increased productivity of microalgae cultivation on these substrates [47,53]. By the way, considering the rather low acetic acid concentration in the different digestates (<100 mg/L in dA and dB, <20 mg/L in dC) and the dilutions applied, acetic acid had likely a minor impact on microalgae growth when using diluted dA, dB and dC. Substrates dA and dB can only partially sustain algal growth when diluted, as both cell density and dry weight of the biomass harvested after growth of *C. vulgaris* and *Scenedesmus I* in liquid medium were strongly reduced. Moreover, it is interesting to note that the total biomass harvested growing *C. vulgaris* and *Scenedesmus I* in dA30W or dB5W is higher compared to previous results achieved by growing *C. sorokiniana* or *Scenedesmus* strains in diluted anaerobic digestate originating from cattle manure digestion or wastewater treatment, obtaining a final biomass concentration in the range of 0.25−0.36 g/L [39,54]. Although the results presented herein are lower compared to other reports where the cultivation of *Chlorella* strains on anaerobic digestates were performed with bubbling 3% CO_2_ in the photobioreactors yielding biomass concentrations of up to 2 g/L [55], in this work the photobioreactors were bubbled with air. It is important also to consider that the anaerobic digestates analyzed here were autoclaved before use, since the main focus of this research work was to investigate if the nutrient composition of the different substrates was sufficient to sustain microalgal cultivation. The presence of competing heterotrophic bacteria could reduce the biomass productivity in photobioreactors, even if the impact of such contaminants strongly depends on the overall cultivation system design as presence and effectiveness of filtering procedures or microalgae concentration in continuous or semicontinous systems.

dA and dB digestates induced a strong accumulation of lipids and carotenoids, redirecting the metabolism as previously observed in several algae species under nutritional stress [12,56]. It is important to note that these substrates were characterized by a high nitrogen content, even when extremely diluted. However, for both dA30W and dB5W, a low phosphate level may be the cause for the limitation in biomass accumulation. In both cases, the dilution applied led to a limitation of phosphorus availability, with phosphate concentrations of ~2.8 and 6 mg/L for d30W and dB5W, respectively; these concentrations are in the range where biomass accumulation is limited by phosphorus availability and close or even lower compared to the minimum phosphate concentration (4.56 mg/L) previously reported to be required in the case of *Chlorella pyrenoidosa* [57]. *C. vulgaris* and *Scenedesmus I* cells growing in dA30W or dB5w are thus under nutritional stress, inducing lipids and carotenoid accumulation, as previously reported. In this condition, carotenoid biogenesis was boosted in both *C. vulgaris* and *Scenedesmus* *I*, while lipid accumulation was increased in *C. vulgaris* only. Carotenoid accumulation is indeed a common response of microalgae to different abiotic stresses, including high light, salinity or nutrient starvation [56,58]. In the case of diluted dA and dB, salinity is comparable or even lower than in the HS medium, and the light intensity applied was the same for all the different photobioreactors. Nutritional stress in phosphate limitation is again the more likely reason for increased carotenoid accumulation observed in cells grown in dA30W or dB5W. Lipid and carotenoid increased accumulation could also be related to other stresses which are differently sensed by different microalgae species, such as sulfate deficiency, which was previously shown to increase lipid accumulation in the case of *C. reinhardtii* [59], or high pH value in the case of dA30W. Increased alkalinity was indeed reported to accelerate lipid accumulation in different microalgae species [60,61] and could be reason for the increased lipid productivity observed in *C. vulgaris.* In addition, in the case of d30W, the air bubbling could have induced nitrogen stripping form the culture due to increased pH value during cultivation (9.3 at the end), increasing the probability of ammonia stripping [62]. The increase of pH value observed in dA30W is likely related to the high alkalinity observed in this digestate (Table 2) and to the stripping of CO_2_ induced by air bubbling. The results obtained highlight how lipid biosynthesis induction is differentially influenced by pH, nitrogen, phosphorus levels and the nitrogen/phosphorous ratio in different species, with the best result obtained in the case of *C. vulgaris* grown in substrate dA30W. Additionally, it is important to note that dA should be used after a strong dilution due to the high turbidity of this digestate (Figure S2).

Other macromolecules with high economic importance that can be obtained by microalgal cultivation are proteins. Protein accumulation per liter of culture was reduced in the case of *C. vulgaris* grown in dA30W or dB5W compared to HS, though it increased when dC was added to HS (Figure 4). Protein accumulation in the case of *C. vulgaris* reflects the accumulation of biomass, since the percentage of protein composition of the dry biomass harvested was essentially unchanged (11.9%−13.7%). However, in the case of *Scenedesmus I*, the percentage of harvested biomass as proteins changed from 10.8% in the case of dC5HS to 24.7% and 31.1% in the case of dA30W and dB5W, respectively, compared to the 18.2% observed for HS. Moreover, the overall protein accumulation in *Scenedesmus I* culture per volume of culture was always around 0.2 g/L. These results suggest that, in the case of *Scenedesmus I*, the nutritional stress increase also had an effect on protein accumulation. The lipid biosynthesis, on the other hand, was mainly boosted in *C. vulgaris*, even if only in the latter did cultivation of microalgae dB give a substantially increased yield of high value products (lipids in this case) per volume of culture. An increase of at least 300% of lipids recovered per volume of culture was indeed observed for *C. vulgaris* grown in dA30W or dB5W.

In addition, it is important to mention that protein or carotenoid production for animal or human nutrition purposes is more difficult when using a digestate from waste treatment considering the necessary certification required and the origin of the digestates. Conversely, the use of microalgae-derived biomass for biofuels production requires less stringent certification, easing the use of the aforementioned digestates. The high lipid production observed using dA or dB points to use of this substrate for algal cultivation for the production of biodiesel. For example, it is possible to hypothesize a first step of growth adding nutrients to dC to achieve high biomass accumulation and a second step of cultivation growing algae in a dA or dB properly diluted with water in order to boost lipid accumulation. Alternatively, harvested algal biomass, or the residues after lipid extraction, can be converted into biogas through anaerobic digestion in a circular process by which anaerobic digestates are recycled to sustain microalgal cultivation. Previous reports demonstrated the relatively high efficiency of anaerobic digestion of microalgae biomass for biogas production by anaerobic digestion [2,63,64,65,66,67,68,69,70]. This possible exploitation of microalgae biomass has the advantage of being based on a technology that is already available and widespread. One last possible scenario is represented by cogeneration plants at a small or medium scale, able to produce heat and energy from biomass combustion.

## 4. Materials and Methods

### 4.1. Digestate Production

#### 4.1.1. Substrate dA: Anaerobic Digestate from Agro-Waste

**Figure 6:**
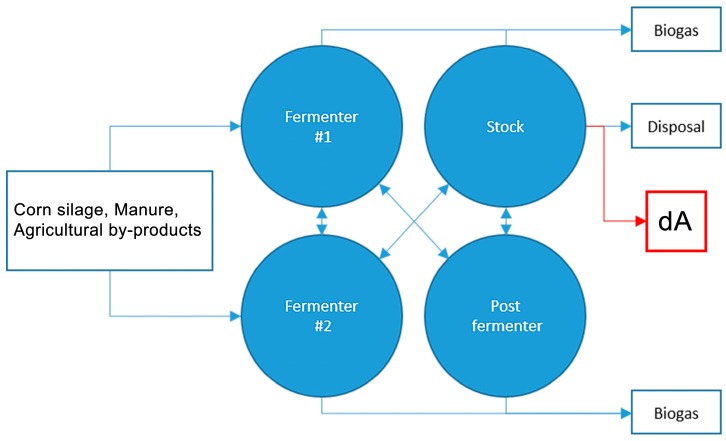
Schematic view of treatment plant producing digestate dA.

The substrate referred to as dA derives from the treatment of livestock effluents originated by the biogas producing plant described in (Figure 6) located at the site of Ca’ Bianca Agricultural Company LTD in Isola della Scala (Verona, Italy). The plant consists of two pre-fermenters, one post-fermenter and a storage tank (warmed and mixed) in which organic matter is anaerobically digested with the concomitant production of biogas. The residual digestate (dA) looks like a thick brown liquid.

#### 4.1.2. Substrate dB: Anaerobic Digestate from Sewage Sludge

**Figure 7:**
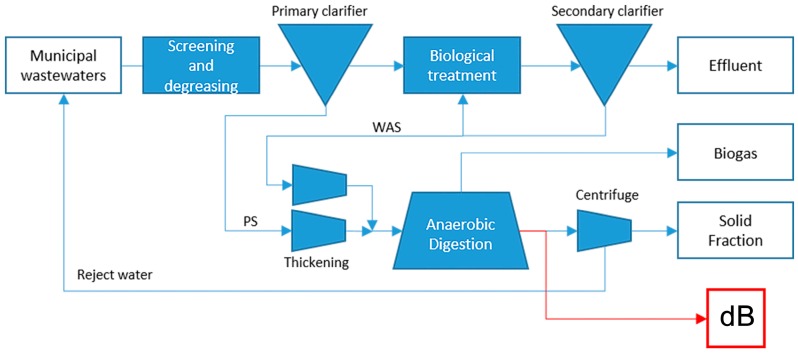
Schematic view of treatment plant producing digestate dB.

Substrate B were collected from the municipal wastewater treatment plant (WWTP) of Verona (Veneto Region, Italy) which has a design treatment capacity of 300,000 PE (Person Equivalent, which indicates a 60 g biochemical oxygen demand in five days for biological degradation of organic matter). The treatment plant is schematically represented in (Figure 7) with an anaerobic digestion of 400−450 m^3^/day of primary and waste-activated sludge (PS and WAS) at mesophilic conditions (37 °C): the resultant sewage sludge from the primary sedimentation of the wastewater and its further biological treatment are thickened and anaerobically digested, determining biogas production. The hydraulic retention time (HRT) of the anerobic digestor is mantained between 22 to 25 days. The PS and the WAS are separately thickened up to 30−58 g/L of total solids by dynamic thickening (Klein Technical Solutions, Niederfischbach, Germany) and a screw drum (Huber, Screw Thickener RoS 2, Munich, Germany), respectively. Substrate dB is collected downstream of this process and it is presented as a turbid liquid.

#### 4.1.3. Substrate dC: Anaerobic Effluent from Municipal Wastewater and Organic Waste

**Figure 8:**
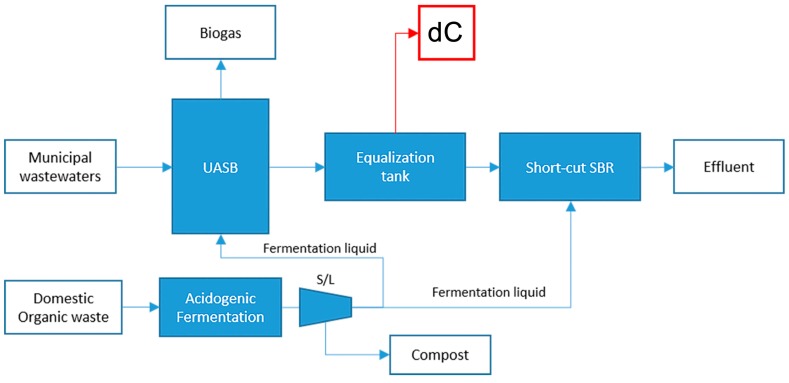
Schematic view of treatment plant producing digestate dC.

The pilot plant in (Figure 8) simultaneously carries out municipal wastewater treatment and disposal of organic fraction of municipal waste disposal. Substrate dC is collected downstream of the anaerobic treatment accomplished by UASB reactor (upflow anaerobic sludge blanket). This kind of reactor relies on the presence of an active granular sludge bed that works as a filter and actively treats water entering from the bottom, while the organic fraction of the municipal solid waste is fermented for the production of readily biodegradable COD [33]. In addition, around 50% of the daily fermentation liquid produced was fed to the UASB reactor in order to boost the biogas production. The final effluent was sampled from a buffer tank after around 2 days of HRT and then stored in a fridge at 2 °C.

The determination of chemical–physical parameters was carried out according to the “Standard Methods for the Examination of Water and Wastewater Characterization” [71,72]. In order to use dA, dB and dC for microalgal cultivation, the digestates were first centrifuged using a Beckman Coulter Avanti JE HPC centrifuge (1760 rcf, 10 min) in order to separate the precipitate from the liquid medium. Digestate was then filtered at 2 mm to remove coarse material as straw or stones and then stored at 4 °C. Absorption of digestates at 440 or 680 nm were measured after centrifugation and filtration using an Aminco DW200 spectrophotometer (SLM instruments, Inc., Urbana, IL, USA).

### 4.2. Microalgal Cultivation

*C. sorokiniana* and *C. vulgaris* strains were obtained, respectively, from the UTEX Culture Collection (www.utex.org) as strain UTEX1230 and from the Culture Collection of Algae at the University of Göttingen, Germany (SAG) as strain 211-11p UTEX. *N. gaditana* strain was kindly gifted by Prof. Tomas Morosinotto from the University of Padua [73]. *Scenedesmus I* and *II* strains were collected in Verona at the production site of the company Algain Energy srl.

Different strains were grown for maintainance mixtrophically in solid TAP medium [45] or, in the case of *N. gaditana*, in F/2 medium [74] in plates at 25 °C, 70 μmol·photons·m^−2^·s^−1^, with a photoperiod of 16:8 h light:dark. Irradiance was provided by warm-white fluorescent lamps.

Spot test on solid medium was performed inoculating different amounts of cells on agar plates. In particular, HS medium and dA, dB and dC digestate diluted 5, 10 or 30 times in deionized water or in HS medium [43] modified as described in [44] were used as growth media for the spot test, adding in each case 15% of agar (*w*/*v*). Only in the case of dC was the undiluted digestate also tested. The different growth media were autoclaved (20 min at 120 °C) before use. After cell inoculation, the plates were kept at 70 μmol·photons·m^−2^·s^−1^ for 30 days. The independent biological replicates in the spot test were 2, each one inoculated at 3 different dilutions (5 µL of cell culture concentrated at 10^6^, 10^5^, 10^4^ cell/mL).

Growth in liquid medium was performed using a MultiCultivator OD-1000 system from Photon Systems Instruments (http://www.psi.cz/products/photobioreactors/multi-cultivator-mc-1000) as described in [75]. The instrument is composed of 8 tubes immersed in a thermostatic bath. Each tube is lit independently by an LED array which in this case was set for all tubes at 400 µmol·m^−2^·s^−1^. A bubbling system avoids cell aggregation. The instrument has been set to measure automatically every 5 min absorption values at 680 nm (proportional to chlorophyll content) and 730 nm (OD, cells number). HS, dA diluted 30 times in deionized water (dA30W), dB diluted 5 times in deionized water (d5W), and dC diluted 5 times in HS (dC5HS) were used as growth media, which were autoclaved before use. Four independent biological replicates were cultivated in closed photobioreactors. Cell density and dry weight were measured as described in [75].

### 4.3. Fluorescence Measurement

Fv/Fm parameter was measured on whole cells previously dark adapted for 20 min. Fv/Fm measurements were performed using a video-imaging system as in [76,77].

### 4.4. Protein Quantification

Protein content has been determined whit QuantiPro BCA assay kit as reported in [78].

### 4.5. Nile Red Assay

Nile red assay was performed in whole cells as described in [79].

### 4.6. Pigment Extraction

Pigments were extracted from microalgae strains as described in [80]. Pigment extracts were then analyzed by HPLC as described in [44].

## Figures and Tables

**Table 1:** Composition of selected digestates.

Parameter	dA	dB	dC	HS
TS (g/L)	94 ± 5	28 ± 3	<0.1	2.1 ± 0.1
TVS (g/L)	65 ± 2	15 ± 3	<0.1	<LOD
COD (gCOD/L)	74 ± 8	18 ± 4	<0.1	<LOD
Total Nitrogen (mgN/L)	5218 ± 314	2750 ± 112	37 ± 6	113 ± 5
VFA (acetic acid, mg/L)	<100	<100	<20	<LOD
Ammonia (mgN/L)	2949 ± 197	1450 ± 65	35 ± 5	113 ± 5
Total Phosphorus (mgP/L)	4870 ± 390	480 ± 44	6.5 ± 0.9	436 ± 15
Orthophosphate (mgP/L)	85 ± 25	30.5 ± 5.2	6.2 ± 0.8	436 ± 15
CaCO_3_ (mg/L)	13,020 ± 750	3780 ± 227	406 ± 54	360 ± 18
pH	8.2 ± 0.1	7.9 ± 0.2	7.6 ± 0.5	6.9 ± 0.1
Conductivity (mS/cm)	26 ± 3	5.4 ±0.9	0.4 ± 0.1	3.2 ± 0.1
OD 440 nm (liquid phase)	13,673 ± 0.05	0.459 ± 0.02	0.0137 ± 0.01	0.002 ± 0.01
OD 680 nm (liquid phase)	2340 ± 0.02	0.221 ± 0.01	0.007 ± 0.01	0.004 ± 0.01

TS: total solid content; TVS: total volatile solid content; COD: chemical oxygen demand; OD: optical density; LOD: limit of detection; VFA: volatile fatty acids.

**Table 2:** HPLC results of pigments extracted from microalgae cultures.

Strain	Growth Medium	Chl a/Chl b	Car/Chl	Car Per 100 Chl
Sce I	**HS**	1.74	1.25	79.86
	**dA30W**	1.67	0.81	123.28
	**dB5W**	0.73	0.68	147.63
	**dC5HS**	2.84	2.62	38.14
Cv	**HS**	4.02	1.58	63.43
	**dA30W**	2.22	0.56	177.96
	**dB5W**	1.47	0.62	160.56
	**dC5HS**	3.80	1.87	53.60

Chl a, b: chlorophyll a, b; Car: carotenoids; Sce I: *Scenedesmus I*; Cv: *Chlorella vulgaris.*

## Supplementary Materials

Supplementary materials can be found at www.mdpi.com/1422-0067/17/10/1692/s1.
