# Supplementary material for: Microalgae Cultivation on Anaerobic Digestate of Municipal Wastewater, Sewage Sludge and Agro-Waste
Source: Int J Mol Sci. 2016 Oct 10;17(10):1692. doi: 10.3390/ijms17101692 (PMC5085724; doi:10.3390/ijms17101692)
Supplement: Supplementary file 1 [file ijms-17-01692-s001.pdf]

Supplementary Materials: Microalgae Cultivation on Anaerobic Digestate of Municipal Wastewater, Sewage Sludge and Agro-Waste

Luca Zuliani, Nicola Frison, Aleksandra Jelic, Francesco Fatone, David Bolzonella and Matteo Ballottari

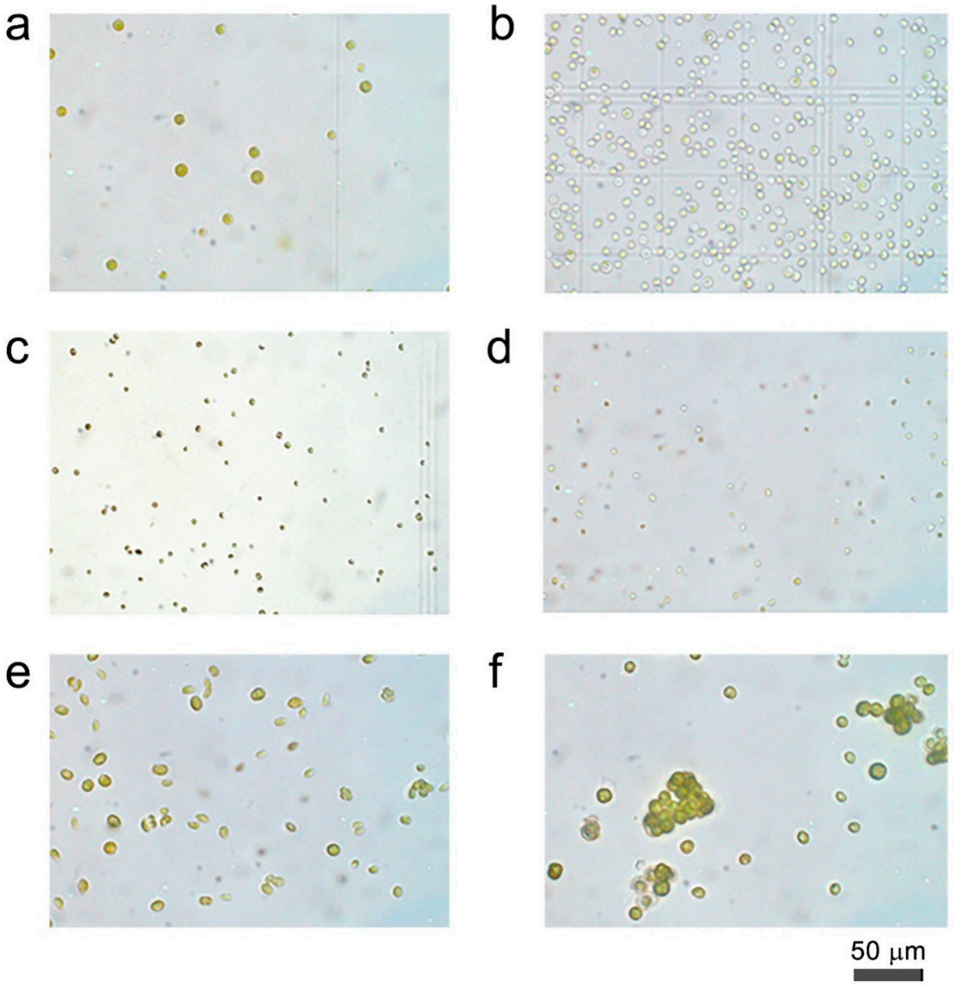

Figure S1. Microscope pictures of the different microalgae herein analyzed. (**a**) *C. reinhardtii*; (**b**) *C. vulgaris*; (**c**) *C. sorokiniana*; (**d**) *N. gaditana*; (**e**) *Scenedesmus I*; (**f**) *Scenedesmus II*. Magnification used was 40×.

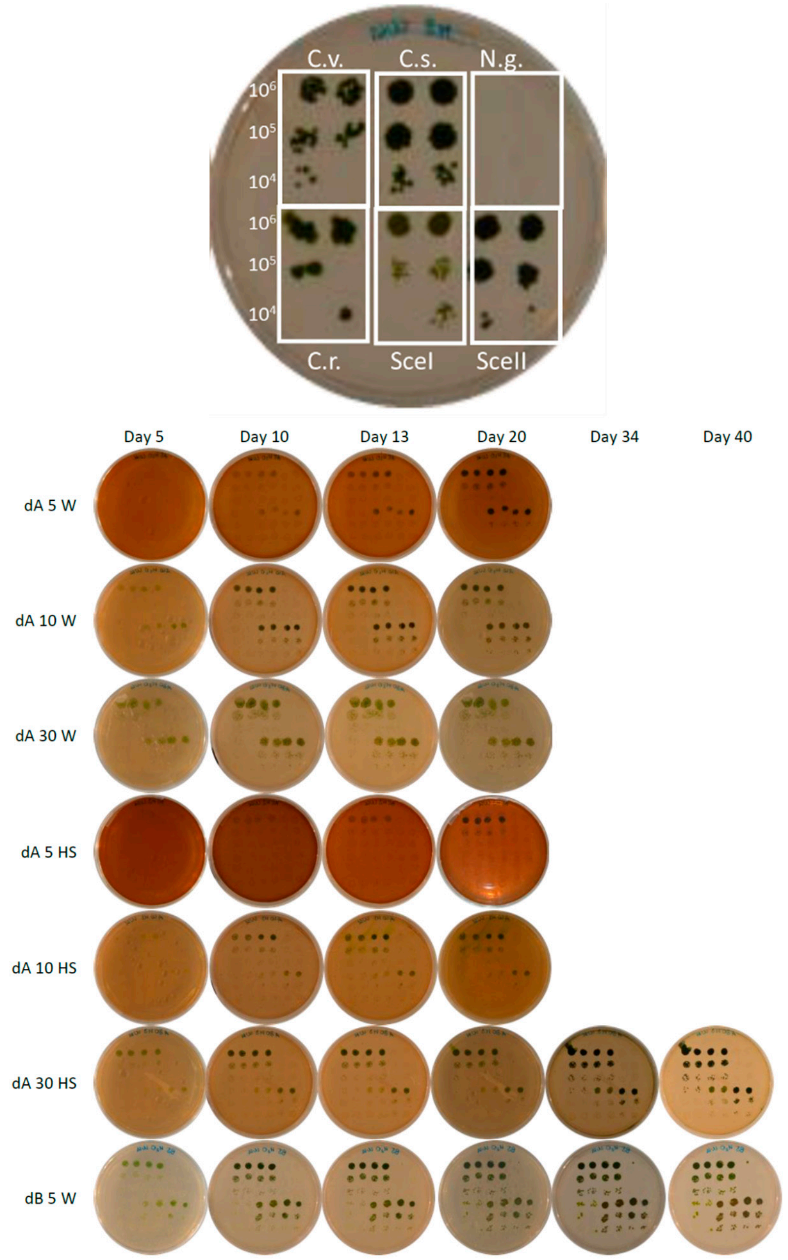

Figure S2. *Cont.*

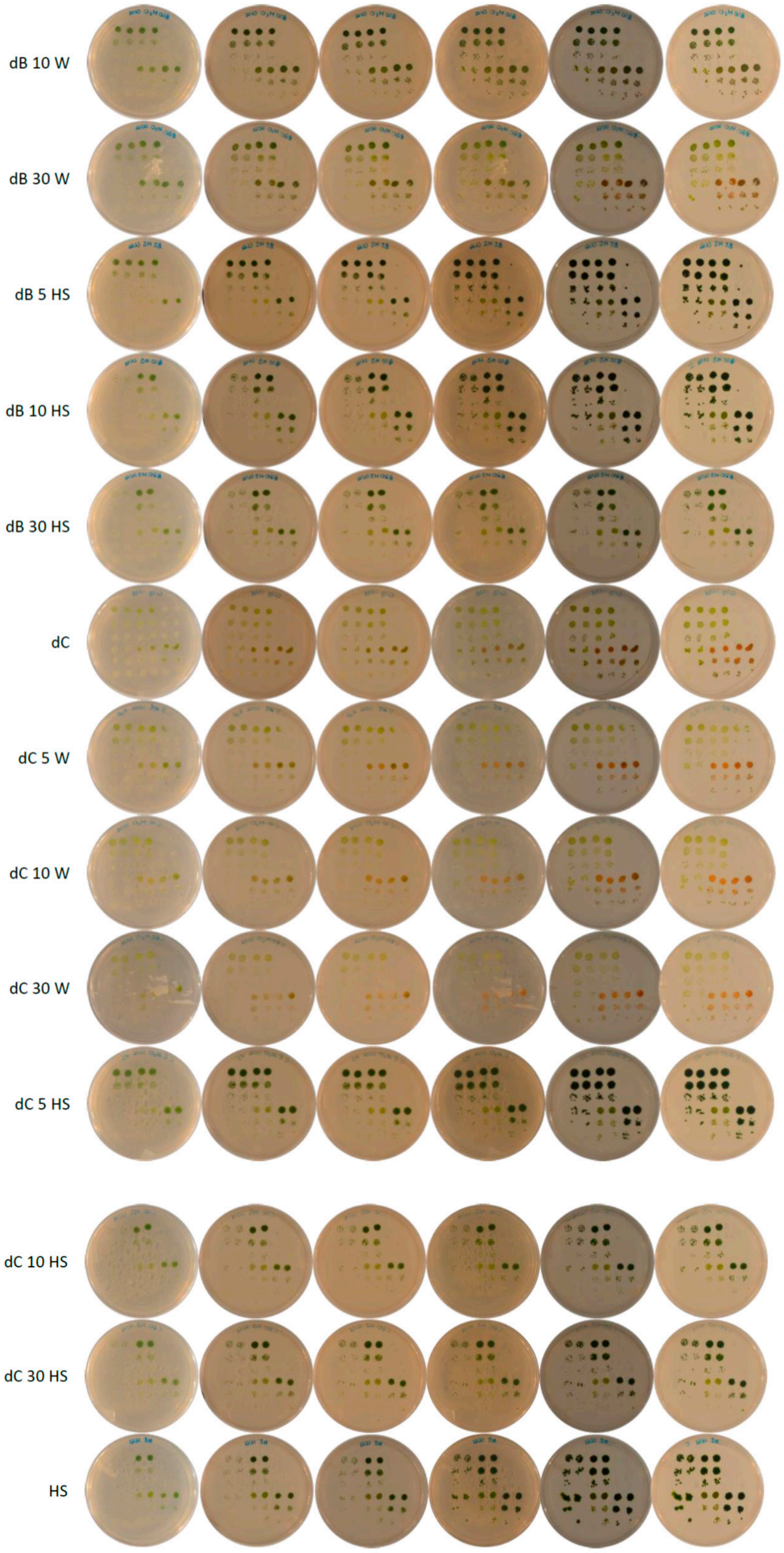

Figure S2. Growth of different microalgal species in solid medium in presence of dA, dB and dC digestates undiluted or dilute 5, 10 or 30 in water (W) or HS medium (HS). Different microalgal species (C.v., *Chlorella vulgaris*; C.s., *Chlorella sorokiniana*; N.g., *Nannochloropsis gaditana*; C.r.; *Chlamydomonas reinhardtii*; SceI, *Scenedesmsus I*; SceII, *Scenedesmus II*) were spotted on solid medium (5 μL of cell concentration of 10^6, 10^5, 10^4 cell/mL) as reported below.
